# CD95-induced osteoarthritic chondrocyte apoptosis and necrosis: dependency on p38 mitogen-activated protein kinase

**DOI:** 10.1186/ar1891

**Published:** 2006-01-16

**Authors:** Lei Wei, Xiao-juan Sun, Zhengke Wang, Qian Chen

**Affiliations:** 1 Department of Orthopaedics, Brown Medical School/Rhode Island Hospital, Providence, Rhode Island, USA

## Abstract

One of the hallmarks of osteoarthritic cartilage is the loss of chondrocyte cellularity due to cell death. However, considerable controversy has recently arisen surrounding the extent of apoptotic cell death involved in development of osteoarthritis (OA). To shed light on this issue, we characterized cell death in primary OA chondrocytes mediated by the CD95 (Fas) pathway. Treatment of chondrocytes with anti-CD95 not only increased the rate of cell death but also increased the production of CD95 ligand by chondrocytes. This reveals a novel autocrine regulatory loop whereby activated chondrocytes may amplify CD95 signals by inducing synthesis of CD95 ligand. Multiple morphologic detection analyses indicated that apoptosis accounted for only a portion of chondrocyte death, whereas the other chondrocytes died by necrosis. Both chondrocyte apoptosis and necrosis depended on the activity of p38 mitogen-activated protein kinase (MAPK) within chondrocytes. Treatment of chondrocytes with the p38 MAPK inhibitor SB203580 abolished anti-CD95 induced cell death by inhibiting the activities of activating transcription factor-2 and caspase-3. In addition, inhibition of p38 MAPK activity in chondrocytes stimulated chondrocyte proliferation, as indicated by 5-bromo-2-deoxyuridine (BrdU) index. Thus, p38 MAPK is a potential therapeutic target, inhibition of which may maintain the cellularity of articular chondrocytes by inhibiting cell death and its amplification signal and by increasing cell proliferation.

## Introduction

Chondrocytes are the only cells in articular cartilage, and thus they are responsible for its structural integrity by maintaining its extracellular matrix. Osteoarthritis (OA) is characterized by destruction of extracellular matrix and loss of chondrocyte function. Chondrocyte depletion was found to be a persistent and important event in OA [1-3], and apoptosis was believed to be a major cause of such cell depletion [4-6]. However, in a recent study [7], although a significant increase in lacunar emptying was observed in human OA cartilage, apoptotic cell death could not fully account for the loss of cells in lacunae. This raises an important question regarding the extent of the contribution of apoptotic cell death to the loss of chondrocytes during OA progression. If apoptosis does not fully account for cell loss in OA cartilage, then what else is involved? More importantly, what are the mechanisms that underlie such loss of chondrocytes? The present study was designed to address these questions by characterizing cell death in primary OA chondrocytes induced by activation of CD95 (Fas).

A significant amount of CD95 ligand (CD95L) has been found in synovial fluid from patients with OA and those with rheumatoid arthritis [8]. Furthermore, in human articular cartilage, CD95 expression in close proximity to OA lesions was found to be increased relative to that further from the lesion [9]. Expression of CD95 and CD95L was higher in aged cartilage than in mature cartilage, which correlated with the decrease in viable cell density in rabbit articular cartilage during aging [10]. This *in vivo *evidence suggests an important role for CD95 in joint cartilage degeneration, although the precise mechanisms are unclear.

p38 Mitogen-activated protein kinase (MAPK) belongs to a family of stress kinases that are activated by proinflammatory cytokines and environmental stresses including altered osmolarity, nutrient deficiency, increased mechanical loading, and decreased oxygen tension [11,12]. Some of these conditions occur readily in OA cartilage. Activated p38 in turn phosphorylates transcriptional factors, thereby transducing signals into the nucleus to alter gene expression [13]. We previously showed that p38 MAPK is essential for regulating hypertrophy and apoptosis in growth plate chondrocytes during endochondral ossification [14]. Because articular chondrocytes may recapitulate hypertrophic processes during OA development, in this study we determined whether p38 activity in human OA chondrocytes plays a role in regulating chondrocyte death. Our findings indicate that there is a strong association between p38 MAPK activity and cell death in human OA chondrocytes. Thus, the p38 MAPK pathway is of potential therapeutic importance as a target for prevention or treatment of chondrocyte loss in OA.

## Materials and methods

### Chondrocyte isolation and primary culture

The study was approved by the institutional review board (approval #0004-03). OA articular cartilage was obtained during total knee replacement surgery. Cartilage slices from normal appearing portions of the tibia plateau were removed and washed in Dulbecco's modified Eagle's medium (DMEM). Chondrocytes were isolated from cartilage as previously described [15]. Briefly, small pieces of cartilage were minced with a scalpel and digested with pronase (2 mg/ml; Boehringer Roche, Indianapolis, IN, USA;) in Hank's balanced salt solution for 30 minutes at 37°C subjected to shaking. After digestion solution was removed, tissue pieces were washed once with DMEM and digested with crude bacterial collagenase (type IA, C 2674; 1 mg/ml; Sigma, Saint Louis, MO USA.) for 6–8 hours at 37°C subjected to shaking. The enzyme reaction was stopped by adding DMEM containing 10% fetal bovine serum. Residual multicellular aggregates were removed by filtration and the cells were washed three times in DMEM.

Chondrocytes were incubated in DMEM containing 10% fetal calf serum, l-glutamine and antibiotics, and were allowed to attach to the surface of the culture dishes. For use in the experiments, cells were trypsinized, washed once, and plated either in eight-well chamber (Nalge Nunc International Corp., Naperville, IL, USA) at 1 × 10^5 ^cells/well or in 100 mm diameter culture dishes (Becton Dickinson Labware, Franklin Lakes, NJ, USA) at 1 × 10^6 ^cells/plate. At 90% confluence, cells were cultured under serum-free conditions overnight before treatment with a mAb anti-Fas CH 11 (100 ng/ml; Panvera, Madison, WI, USA) in serum-free medium for 17 hours, or with SB203580 (10 μmol/l) for 2 hours before anti-Fas treatment. Control cells were treated with either dimethyl sulphoxide or a mouse isotype control antibody IgM (M 5909; Sigma), as indicated.

### Measurement of cell death

After chondrocytes were stimulated as indicated, supernatants containing floating cells were harvested and adherent cells were scratched off the plate with a disposable cell lifter. Cells were combined, spun down, and washed with phosphate-buffered saline (PBS). Cell viability was analyzed by trypan blue dye exclusion assays. Apoptotic cells were detected by *in situ *cell death fluorescein detection kit (terminal dUTP nick-end labeling [TUNEL]; Boehringer Mannheim) and quantified by flow cytometry. Briefly, collected cells were washed twice with PBS containing 1% bovine serum albumin at 4°C. Cell suspensions were fixed with 100 μl freshly prepared paraformaldehyde solution (4% in PBS; pH 7.4) for one hour at room temperature, followed by centrifugation to remove fixative. Cells were washed once with 200 μl PBS, resuspended in 100 μl permeabilization solution (0.1% triton X-100 in 0.1% sodium citrate) for two minutes on ice. After cells were washed two times with 200 μl PBS, they were resuspended in 50 μl TUNEL reaction mixture or in 50 μl label solution as a negative control. Cells were incubated for 30 minutes at 37°C in a humidified atmosphere in the dark, before apoptotic cells were quantified by fluorescence-activated cell sorter (FACS) analysis. Positive control of apoptosis was generated by incubating cells with DNase I (grade I; 0.5 mg/ml) in 50 mmol/l Tris-HCl (pH 7.5), 1 mmol/l MgCl_2_, and 1 mg/ml bovine serum albumin for 10 min at room temperature before the TUNEL labeling reactions.

Apoptotic and necrotic cells were also analyzed with an Annexin-V-Fluos Staining Kit (Roche Molecular Biochemicals, Indianapolis, IN, USA). Cells at early stages of apoptosis were labeled by annexin V, whereas necrotic cells were labeled by propidium iodide, which permeated them and stained their nuclei. About 1 × 10^6 ^cells were incubated with fluorescein isothiocyanate (FITC)-labeled annexin V and propidium iodide simultaneously, before quantification by FACS analysis.

### Immunocytochemistry

Immunocytochemical analyses of chondrocyte phenotype were performed as described previously [16]. using anti-type I and anti-type II collagen mAb (Chemicon International, Temecula, CA, USA). A secondary antibody, rhodamine-conjugated donkey anti-mouse IgG (H+L; Jackson ImmunoResearch Laboratories, Inc. West Grove, PA, USA), was diluted 1:500 in PBS containing 1% bovine serum albumin. Slides were mounted in FluorSave™ reagent (Calbiochem-Novabiochem Corporation, La Jolla, CA, USA) and viewed under a fluorescent microscope (Nikon microscope E 800).

### Histochemistry

Pieces of cartilage from normal appearing areas of the OA-affected tibial plateaus were collected and fixed in 4% paraformaldehyde before they were embedded in tissue freezing medium and processed for cryostat section. Sections (5 μm thick) were cut perpendicular to the cartilage surface. Distribution of Fas antigen in cartilage was examined by immunohistochemistry with a Histostain SP kit (Zymed, San Francisco, CA, USA) using anti-Fas mAb Ch-11 (Panvera) as primary antibody. Sections were fixed at -20°C with 70% ethanol and 50 mmol/l glycine (pH 2.0) for 20 minutes, treated with hyaluronidase (2 mg/ml; Sigma Chemical Co., St Louis, MO) for 30 minutes at 37°C, and incubated in 0.2% Triton X-100/PBS for 5 minutes at room temperature. Slides were washed with PBS and treated with peroxidase quenching solution to eliminate endogenous peroxidase activity. Sections were then incubated with primary antibodies for 1 hour at 37°C followed by biotinylated secondary antibodies for 10 minutes at room temperature. After washing with PBS, sections were incubated with a streptavidin–peroxidase conjugate for ten minutes at room temperature followed by a solution containing diamino-benzidine (chromogen) and 0.03% hydrogen peroxide for 5 minutes at room temperature. Sections were counterstained with hematoxylin, dehydrated, and mounted. Photography was performed using a Nikon microscope.

### Western blot

Total protein was extracted from cells and quantified as described with BAC Protein Assay Reagent Kit (Pierce, Rockford. IL). For each sample, 10 μg total protein was electrophoresed in 10% SDS PAGE under reducing conditions before blotting and probing with polyclonal antibodies against p38 MAPK (SC535; Santa Cruz, CA, USA), phospho-p38 MAPK (pTGPY, Santa Cruz) and activating transcription factor (ATF)-2 (SC187, Santa Cruz), and a mAb against phospho-ATF-2 (SC8398, Santa Cruz). All of the antibodies were diluted 1:1,000 in PBS-Tween containing 1% bovine serum albumin. Horseradish peroxidase-conjugated goat anti-rabbit or anti-mouse IgG (H+L; Bio-Rad Laboratories, Richmond, CA, USA) were diluted 1:3,000 in PBS-Tween, and used as secondary antibodies. Visualization of immunoreactive proteins was achieved using ECL Western blotting detection reagents (Amersham, Arlington Heights, IL, USA) and by subsequently exposing the membrane to Kodak X-Omat AR film.

### Caspase-3 activity assay

A caspase-3 cellular activity assay kit (BIOMOL^® ^Research Laboratories, Inc., Plymouth Meeting, PA, USA) was used to measure caspase-3 activity, in accordance with the manufacturer's instructions. Briefly, chondrocytes treated with anti-CD95 antibody, SB203580 and anti-CD95 antibody, or SB203580 alone were harvested, washed in PBS, and resuspended in cell lysis buffer. Cytosolic extract was collected from supernatant after centrifugation at 10,000 *g *for ten minutes at 4°C, before it was incubated in microtiter plate with assay buffer. After the reaction was started by the addition of 10 μl Ac-DEVD-pNA substrate (final substrate concentration 200 μmol/l), plate absorbance at 405 nm was read by a microtiter plate reader. Caspase-3 activity was calculated as pmol/min per 2 × 10^6 ^cells.

### Cell proliferation assay

Proliferation of chondrocytes was determined using BrdU Kit I (Roche, Indianapolis, IN, USA), in accordance with the manufacturer's instruction. Briefly, after cells were treated by anti-Fas antibody, SB, or SB-positive anti-Fas antibody, they were incubated with BrdU labeling medium in eight-well chambers for 60 minutes at 37°C in 5% carbon dioxide. Cells were fixed with ethanol fixative for 20 minutes at -20°C, washed, and incubated with anti-BrdU working solution for 30 minutes at 37°C. After incubation with anti-mouse-immunoglobulin-fluorescein working solution for 30 minutes at 37°C in the presence of Hoechest solution (1:1,000), cells were washed and examined in a fluorescence microscope.

### Real-time RT-PCR

Real-time RT-PCR was performed as previously described [11]. Briefly, total RNA was isolated from chondrocytes with RNeasy isolation kit (Qiagen). One microgram of RNA was reverse transcribed using Superscript™ II Rnase H Reverse Transcriptase Kit (Invitrogen, Carlsbad, CA, USA). Of the resulting cDNA, 30 ng/μl was used as the template to quantify the relative content of mRNA by real-time PCR (ABI PRISM 7700 sequence detection system, Applied Biosystems, Foster City, CA USA) using respective primers and SYBR Green. The primers of Fas ligand were designed using Primers Express software (BioTools Incorporated, Edmonton, AB, T5J 3H1, Canada): forward primer sense ACA CCT ATG GAA TTG TCC TGC, and antisense AGT TTC ATT GAT CAC AAG GC. PCR reactions were performed with TaqMan PCR master mix kit (Applied Biosystems, Foster City, CA, USA). The 18S RNA was amplified at the same time and used as an internal control. The cycle threshold values for 18S RNA and that of CD95L were measured and calculated using computer software. Relative transcript levels were calculated as x = 2^-ΔΔCt^, in which ΔΔCt = ΔE - ΔC, and ΔE = Ct_EXP _- Ct_18S_, and ΔC = Ct_CTL _- Ct_18S_.

### Statistical analysis

Statistical analysis was performed by analysis of variance followed by a Tukey's test for multiple comparisons at a rejection level of 5%. Data are expressed as mean ± standard deviation.

## Results

### Expression of CD95 and CD95 ligand

**Figure 1:**
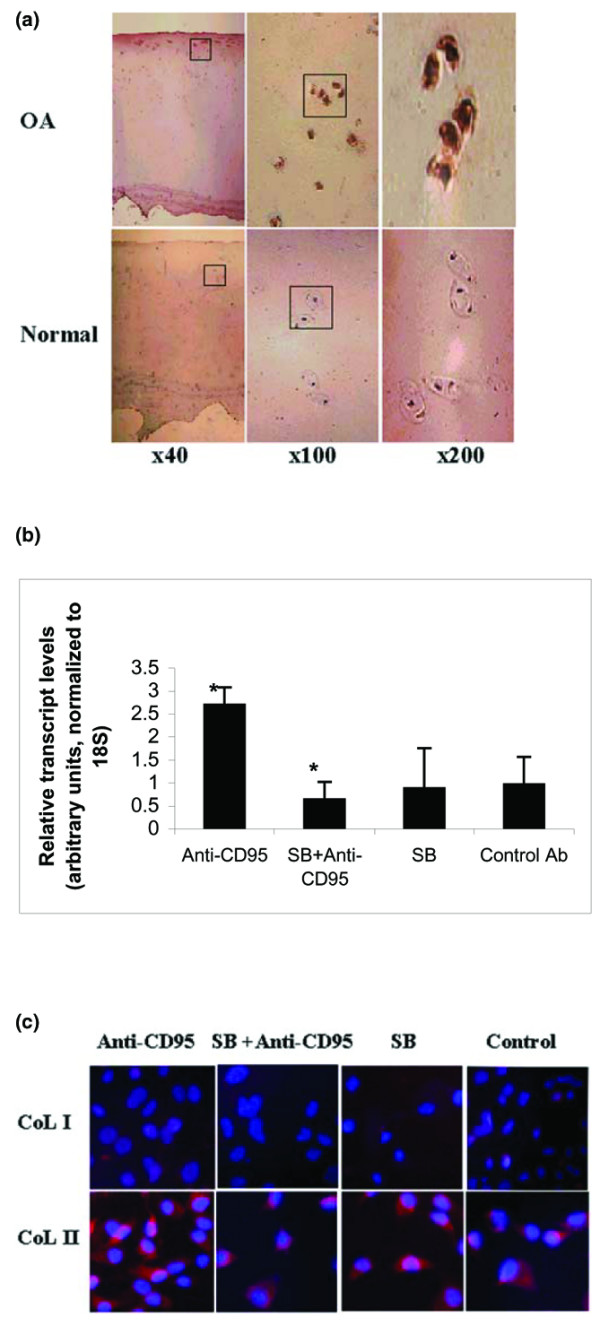
Expression of CD95 and CD95L by OA chondrocytes. **(a) **Micrographs of immunohistochemcal analysis of CD95 expression in normal (bottom panels) and OA cartilage (top panels). Frozen sections from normal and OA articular cartilage were incubated with a monoclonal antibody CH 11 against CD95. Different (original) magnifications are indicated. Results are representative of two normal and four OA cartilage samples. **(b) **mRNA levels of CD95L in primary OA chondrocytes by real-time RT-PCR analysis. Total RNA was isolated from chondrocytes from OA cartilage following treatment as indicated below. *P < 0.05 versus treatment with Control Ab (a mouse isotype control antibody IgM). Each bar represents the mean ± standard deviation of three experiments. OA chondrocytes (donor age 63 years) stained with annexin V and PI. Results are representative of three OA cartilage samples. **(c) **Micrographs of immunocytochemical analysis of collagens type II and type I in OA chondrocytes following treatment as indicated below. Chondrocytes were reacted with rhodomaine mAb against type II or type I collagen, respectively. Chondrocyte nuclei were indicated by Hoechest blue dye staining. Anti-CD95: treatment with a mAb anti-Fas CH 11 (100 ng/ml) in serum-free medium for 17 hours. SB: treatment with SB203580 (10 μmol/l) for 17 hours. SB+Anti-CD95: treatment with SB203580 for 2 hours followed by anti-Fas treatment for 17 hours. Control: cells were treated with DMSO only. Results are representative of 3 OA cartilage samples. CD95L, CD95 ligand; DMSO, dimethyl sulfoxide; mAb, monoclonal antibody; OA, osteoarthritis; PI, propidium iodide.

To determine whether CD95 and its ligand were involved in induction of cell death in cartilage, we examined their expression in chondrocytes. Immunohistochemical analysis indicated that CD95 was expressed in cartilage, especially in the cells from the superficial and middle zones of OA cartilage (Figure 1a). Although CD95L was expressed at low levels in chondrocytes isolated from OA cartilage, its mRNA level was increased more than 2.5-fold in response to anti-CD95 treatment (Figure 1b). Thus, activation of the CD95 pathway induced synthesis of CD95L by chondrocytes. This induction was abolished by treatment of chondrocytes with SB203580, a specific inhibitor of p38 MAPK. Therefore, induction of CD95L in chondrocytes is dependent on p38 MAPK activities. The phenotype of chondrocytes, however, was not affected by the activation of the CD95 pathway or inhibition of p38 MAPK, because they were positive for collagen type II, a marker of chondrocytes, under all treatment conditions (Figure 1c).

### CD95-mediated chondrocyte death

**Figure 2:**
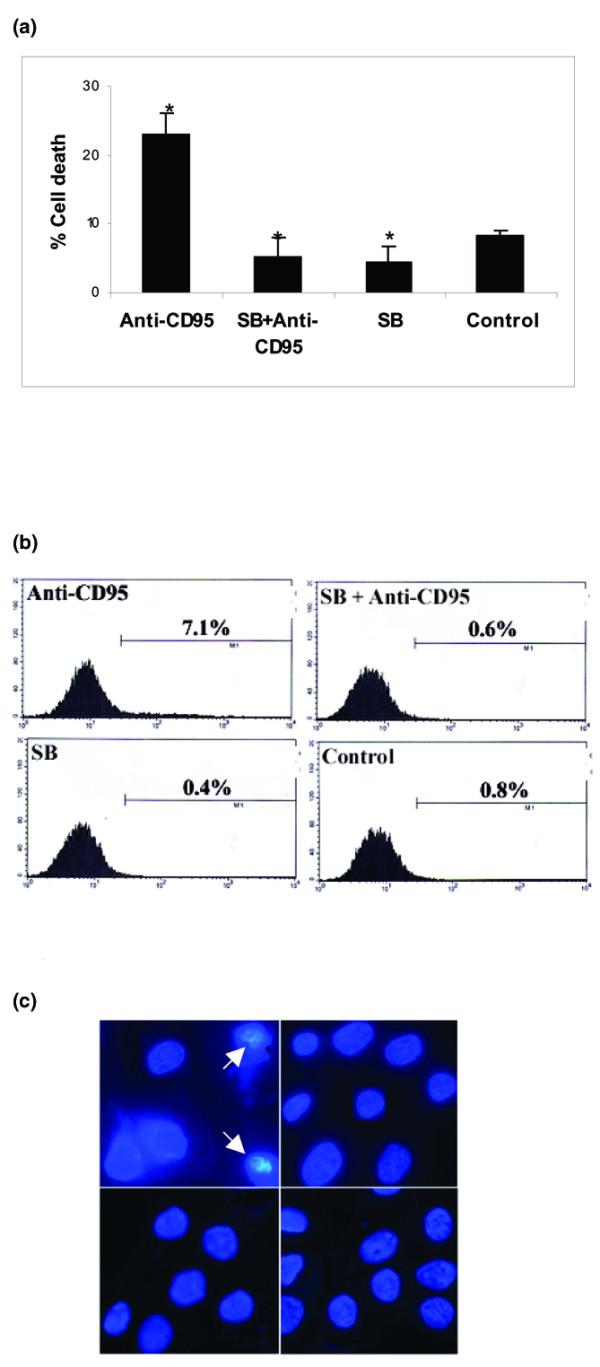
p38 MAPK activity regulates cell death induced by anti-CD95. **(a) **Total cell death rate for OA chondrocytes quantified by trypan blue exclusion assay after treatment of cells as indicated below. *P < 0.05 versus control. The graph shows the average of three independent experiments. **(b) **Apoptosis rate of OA chondrocytes quantified by flow cytometry. Chondrocytes were fluorescently labeled by TUNEL assay after treatment of cells as indicated below. The data shown are representative of three OA cartilage samples. Anti-CD95: treatment with a mAb anti-Fas CH 11 (100 ng/ml) in serum-free medium for 17 hours. SB: treatment with SB203580 (10 μmol/l) for 17 hours. SB+Anti-CD95: treatment with SB203580 for 2 hours followed by anti-Fas treatment for 17 hours. Control: cells were treated with DMSO only. **(c) **Chondrocytes were labeled with Hoechst after treatment of cells as indicated above. Nuclear condensation (indicated by an arrow) was detected in the cells treated with Anti-CD95. DMSO, dimethyl sulfoxide; mAb, monoclonal antibody; MAPK, mitogen-activated protein kinase; OA, osteoarthritis; PI, propidium iodide; TUNEL, terminal dUTP nick-end labeling.

To determine whether activation of the CD95 pathway induced cell death, we quantified cell death rate using trypan blue exclusion assay. In response to anti-CD95 treatment, death rate for chondrocytes increased to 23% from the basal rate of 5–8% (Figure 2a). Inhibition of p38 MAPK activity by treatment of chondrocytes with SB203580 completely inhibited anti-CD95 induced cell death. This indicates that p38 MAPK is a key mediator of anti-CD95 induced chondrocyte death.

To determine whether apoptosis was the underlying mechanism of anti-CD95 induced cell death, apoptotic chondrocytes were labeled with TUNEL and quantified by FACS analysis. Anti-CD95 treatment increased the apoptosis rate to 7% from the basal rate of 0.4–1% (Figure 2b). Apoptotic cell features including nuclear condensation was detected in the cells treated with anti-CD95 (Figure 2c). This increase in apoptosis rate by anti-CD95 was completely inhibited by treatment of chondrocytes with the p38 inhibitor, indicating that CD95 induced chondrocyte apoptosis was also dependent on p38 activity. However, the apoptosis rates (basal rate 0.4–1%, CD95 induced rate 7%) were lower than the total cell death rates (basal rate 5–8%, CD95 induced rate 23%).

### Mechanisms of chondrocyte death include both apoptosis and necrosis

**Figure 3:**
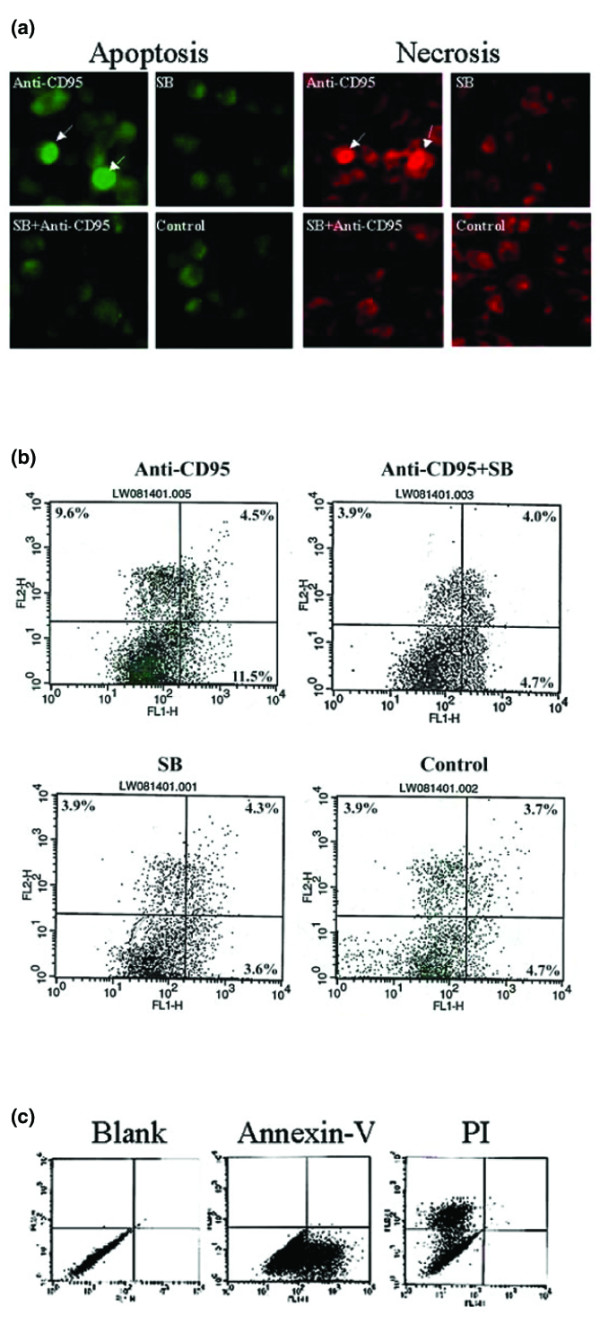
Chondrocyte death consists of apoptosis and necrosis. **(a) **Morphologic analysis of chondrocyte apoptosis and necrosis. The nuclei of apoptotic cells were labeled by TUNEL with green fluorescence whereas the nuclei of necrotic cells were labeled by PI with red fluorescence (donor age 63 years). All cell nuclei were labeled with blue Hoechst dye. Results are representative of three OA cartilage samples. **(b) **The rates of cell apoptosis and necrosis quantified by flow cytometry. Apoptotic cells were labeled by annexin V whereas necrotic cells were labeled by PI at the same time. Total events are 10,000. The x axis represents FL1-H (log) with green fluorchrome for annexin V labeling, and the Y axis represents FL2-H (log) with red fluorchrome for PI labeling. The percentage of cells that are single or double positive for annexin V and PI is indicated in each grid. The upper left grid represents the number of PI single positive cells. The lower right grid represents the number of annexin V single positive cells. The upper right grid represents the number of cells positive for both PI and annexin V. The number of cells that are single positive for PI is 9.6% for anti-CD95, and 3.9% for control, SB, and Anti-CD95+SB. Anti-CD95: treatment with a mAb anti-Fas CH 11 (100 ng/ml) in serum-free medium for 17 hours. SB: treatment with SB203580 (10 μmol/l) for 17 hours. SB+Anti-CD95: treatment with SB203580 for 2 hours followed by anti-Fas treatment for 17 hours. Control: cells were treated with DMSO only. Results are representative of five OA cartilage samples. **(c) **FACS analysis of OA chondrocytes labeled by annexin V and PI distinguishes apoptosis from necrosis. Chondrocytes were labeled into four groups by Annexin-V-FLUOS Staining Kit: x axis is FL1-H (log), and fluorchrome is green for annexin V (panel b [lower right] and panel c [lower right]); and y axis is FL2-H (log), and fluorchrome is red for PI (panel b [upper left] and panel c [upper left]). Living cells are shown to the lower left and double staining to the upper right of panels b and c. DMSO, dimethyl sulfoxide; FACS, fluorescence-activated cell sorter; mAb, monoclonal antibody; OA, osteoarthritis; PI, propidium iodide; TUNEL, terminal dUTP nick-end labeling.

To reconcile the discrepancy between the apoptosis rate and the total cell death rate, we simultaneously labeled the apoptotic nuclei with TUNEL and the necrotic nuclei with propidium iodide. Morphologic analysis indicated that the number of TUNEL or propidium iodide positive cells was increased in anti-CD95 treated chondrocytes (Figure 3a). To quantify the rates of apoptosis and necrosis, we simultaneously labeled chondrocytes with anti-annexin V (a marker of apoptosis) and propidium iodide (a marker of necrosis). FACS analysis indicated that the rate of chondrocyte necrosis was greatly increased by anti-CD95 treatment (Figure 3b). This suggested that necrosis also contributed to chondrocyte death. Both the rate of apoptosis and that of necrosis were increased by anti-CD95 treatment, and these increases were diminished by inhibition of p38 MAPK (Figure 3b). Thus, both CD95 induced chondrocyte apoptosis and necrosis depended on p38 MAPK activity. Because OA chondrocytes comprised distinctive annexin V and propidium iodide labeled populations (Figure 3c), this indicated that chondrocyte death consisted of both apoptosis and necrosis.

### Components of the CD95/p38 pathway in chondrocytes

**Figure 4:**
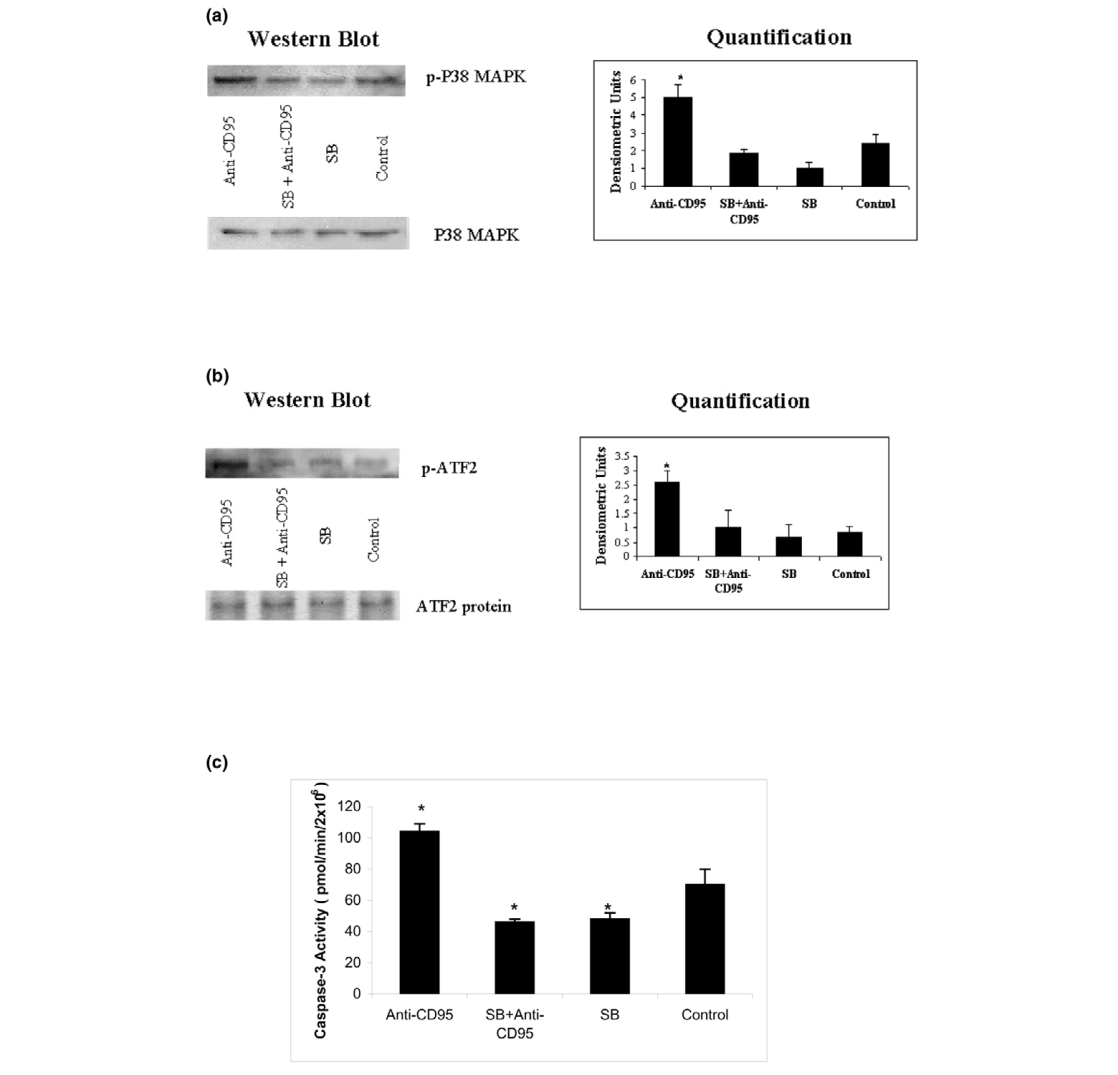
Anti-CD95 induced chondrocyte death is p38 MAPK dependent. **(a) **Western blot analysis of the levels of phosohorylated-p38 MAPK and p38 MAPK protein (left panel) and the quantification of the western blot by densitometric analysis (right panel). *P < 0.05 versus control. Each bar represents mean ± standard deviation of three experiments. **(b) **The levels of phosphorylated ATF-2 and ATF-2 protein were determined by western blot on the left. Quantification of the western blot by densitometric analysis is shown on the right. *P < 0.05 versus control. Each bar represents mean ± standard deviation of three experiments. (c) Caspase-3 activities were determined using a caspase-3 cellular activity assay kit. *P < 0.05 versus control. each bar represents mean ± standard deviation of three experiments. Anti-CD95: treatment with a mAb anti-Fas CH 11 (100 ng/ml) in serum-free medium for 17 hours. SB: treatment with SB203580 (10 μmol/l) for 17 hours. SB+Anti-CD95: treatment with SB203580 for 2 hours followed by anti-Fas treatment for 17 hours. Control: cells were treated with DMSO only. Results are representative of three OA cartilage samples. ATF, activating transcription factor; DMSO, dimethyl sulfoxide; mAb, monoclonal antibody; MAPK, mitogen-activated protein kinase; OA, osteoarthritis.

To further determine the role of p38 MAPK activity in regulating CD95 induced cell death, we quantified p38 MAPK activity in chondrocytes after treatment with anti-CD95 or SB203580, or both. Western blot analysis indicated that anti-CD95 treatment significantly increased p38 activity in chondrocytes, and this increase was abolished by treatment with SB203580 (Figure 4a). Thus, p38 MAPK activity in chondrocytes paralleled the chondrocyte death rate induced by anti-CD95 treatment. This indicated that p38 MAPK activity was a key mediator of CD95 induced chondrocyte death.

Next, we identified potential components of the CD95 pathway in chondrocytes. The first was ATF-2, a putative substrate of p38 MAPK. Activity of ATF-2 was induced by anti-CD95, and this induction was diminished by inhibition of p38 MAPK activity (Figure 4b). Likewise, activity of caspase-3, an executioner enzyme in the apoptosis pathway, was induced by anti-CD95 (Figure 4c). This induction was also abolished by inhibiting p38 MAPK activity in chondrocytes. Thus, both ATF-2 and caspase-3 are potential components of the CD95 pathway downstream of p38 MAPK.

### Suppression of p38 MAPK activity increased chondrocyte proliferation

**Figure 5:**
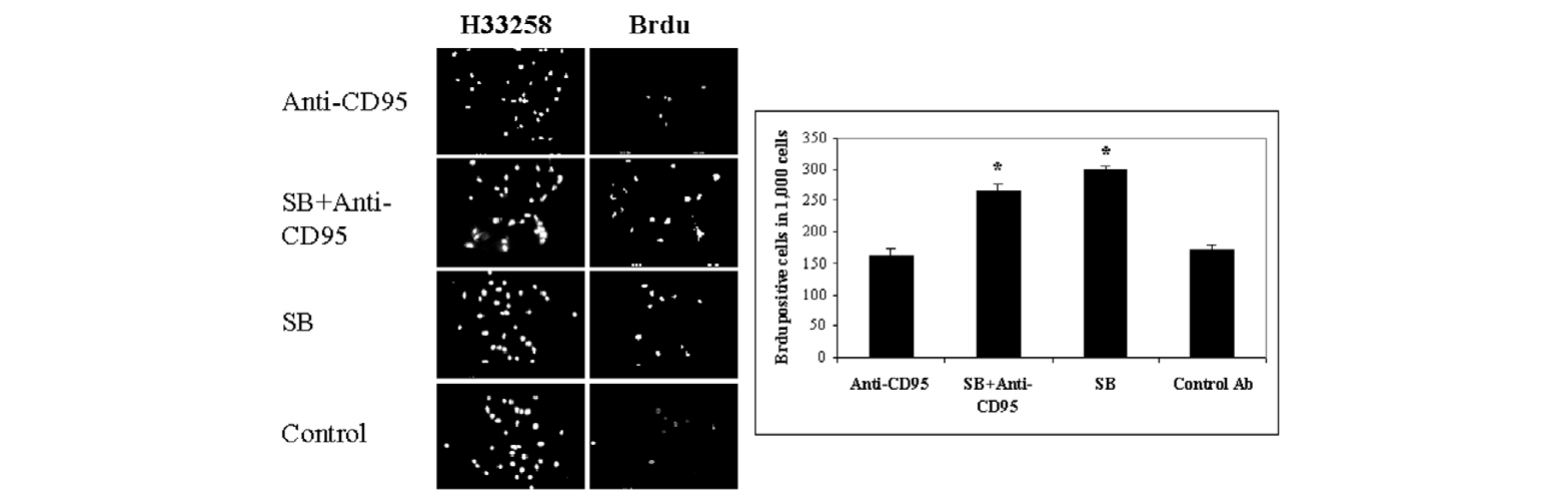
Cell proliferation regulated by p38 MAPK activity. **(a) **The number of BrdU positive cells in 1,000. **(b) **Cell proliferation was evaluated by BrdU staining whereas all cell nuclei were stained by Hoechst 33258 dye. *P < 0.05 versus control. Each bar represents mean ± standard deviation of three experiments. Anti-CD95: treatment with a mAb anti-Fas CH 11 (100 ng/ml) in serum-free medium for 17 hours. SB: treatment with SB203580 (10 μmol/l) for 17 hours. SB+Anti-CD95: treatment with SB203580 for 2 hours followed by anti-Fas treatment for 17 hours. Control Ab: cells were treated with a mouse isotype control antibody IgM. Results are representative of three OA cartilage samples. BrdU, 5-bromo-2-deoxyuridine; mAb, monoclonal antibody; MAPK, mitogen-activated protein kinase; OA, osteoarthritis.

To determine whether inhibition of p38 MAPK activity affected chondrocyte proliferation in addition to its death, we quantified chondrocyte proliferation rate by measuring BrdU labeling index. Although anti-CD95 did not affect chondrocyte proliferation, treatment of SB203580 significantly increased chondrocyte proliferation rate (Figure 5). Therefore, suppression of p38 MAPK activity increased cell proliferation.

## Discussion

Chondrocytes, the only type of cells in cartilage, are responsible for maintaining extracellular matrix in cartilage. The mechanism of cell death is not clear, although an increase in the number of empty lacunae has been found in OA cartilage [2,17]. In the past, major effort has been devoted to the study of apoptosis of OA chondrocytes under the assumption that apoptosis is responsible for chondrocyte death in OA. However, a recent study [7] identified a discrepancy between the rate of lacunar emptying in cartilage and the rate of apoptotic cell death. In that study we found that apoptosis accounted for only a portion of OA chondrocytes committed to cell death; OA chondrocyte death included both apoptosis and necrosis. This observation potentially explains the discrepancy in previous studies between the rate of total cell death, as reflected by lacunar emptying, and the rate of apoptotic death in OA chondrocytes [7].

Necrosis and apoptosis are two major types of cell death. Although not mutually exclusive, they are mechanistically and morphologically distinct types of cell death [18]. In particular, apoptotic cell death is mediated by activation of caspases whereas necrotic cell death is not. In addition, secondary necrosis may occur at the later stages of apoptosis. A combination of various morphologic characterizations, such as labeling chondrocytes with annexin V in the membrane or TUNEL in the nuclei, as performed in the present study, is required to measure the rate of apoptosis accurately. We found that some morphologic analyses such as the trypan blue exclusion assay detected the rate of total cell death, which may include apoptosis and necrosis, rather than apoptosis specifically. In contrast, labeling cells with annexin V and propidium iodide simultaneously followed by dual parameter flow cytometry generated two distinctive populations of apoptotic cells and necrotic cells, respectively.

Based on our findings, we suggest that necrosis is a major form of cell death in OA chondrocytes, in addition to apoptosis. At least two types of factors may account for the occurrence of necrotic cell death in OA. One factor is cytokines such as the CD95L, as shown in the present study. Such factors are readily available in the synovium under inflammatory conditions, which may occur during the pathogenesis of OA [18-20]. This is also consistent with the notion that although apoptosis often affects individual cells without involvement of inflammatory responses, necrosis affects groups of cells in association with an inflammatory response [18]. The second factor is mechanical damage of articular cartilage, which is often associated with OA pathogenesis. It was previously shown that chondrocyte necrosis occurs in impact damaged articular cartilage [21]. Our finding that necrosis is a major form of cell death in OA chondrocytes may have strong implications for devising strategies for prevention and treatment of OA.

We have also shown that activation of the CD95 pathway in chondrocytes increases not only the cell death rate but also the production of CD95L by chondrocytes. The initial induction of cell death in cartilage by activating the CD95 pathway is thought to occur via CD95L derived from the neighboring synovium [22]. However, because the extracellular matrix in cartilage may act as a barrier to prevent diffusion of CD95L into the deep layer of articular cartilage, the contribution of the CD95 pathway to induction of chondrocyte death in OA cartilage was not clear. Our data suggest that CD95 activated chondrocytes elevate their own production of CD95L, which may in turn facilitate the cell death process by sustaining and amplifying death signals. This autocrine regulatory loop may contribute to the catastrophic degeneration cascade that occurs in cartilage once it is activated.

We found that p38 MAPK activity in chondrocytes is essential to induction of cell death by the CD95 pathway. This finding is consistent with previous observations in other types of cells, such as neuronal and lymphoma B cells [23,24]. Furthermore, we identified two components of the CD95/p38 MAPK pathway that may mediate chondrocyte death. The first is ATF-2, which is an important transcription factor and a known substrate of p38 MAPK [25,26]. ATF-2 is involved in regulating chondrocyte proliferation and differentiation because ATF-2 knockout mice exhibit cartilage defects during development [27]. The second component is caspase-3, an executioner caspase that mediates cell apoptosis [28]. Our study suggests that both ATF-2 and caspase-3 function downstream of p38 MAPK in regulating chondrocyte death. Elevation in p38 activity with anti-CD95 induces cell death by stimulating ATF-2 and caspase-3 activity, whereas repression of p38 activity by SB203580 inhibits chondrocyte cell death by depressing ATF-2 and caspase-3 activity. In addition to blocking cell death, inhibiting p38 MAPK activity stimulates chondrocyte proliferation. Similar effects of p38 MAPK on proliferation was observed in other types of cells [13,29]. Because a slow rate of chondrocyte proliferation was observed in OA cartilage [30,31], inhibiting p38 MAPK activity in chondrocytes may increase the cellularity of OA chondrocytes by increasing cell proliferation in addition to decreasing cell death. Our observations *in vitro *using OA chondrocytes have strong implications for our understanding of the mechanisms underlying OA chondrocyte death; however, the effectiveness of such intervention using p38 MAPK inhibitors awaits verification using animal models *in vivo*.

## Conclusion

In the present study we found that cell death induced by anti-CD95 in chondrocytes includes both apoptosis and necrosis during development of OA. Both chondrocyte apoptosis and necrosis depended on the activity of p38 MAPK within chondrocytes. Treatment of chondrocytes with p38 MAPK inhibitor SB203580 abolished anti-CD95 induced cell death by inhibiting the activities of ATF-2 and caspase-3. In addition, inhibition of p38 MAPK activity in chondrocytes stimulated chondrocyte proliferation. Thus, p38 MAPK is a potential therapeutic target, inhibition of which may maintain the cellularity of articular chondrocytes by inhibiting cell death and its amplification signal and by increasing cell proliferation.

## Abbreviations

ATF = activating transcription factor; BrdU = 5-bromo-2-deoxyuridine; CD95L = CD95 ligand; DMEM = Dulbecco's modified Eagle's medium; FACS = fluorescence-activated cell sorter; mAb = monoclonal antibody; MAPK = mitogen-activated protein kinase; OA = osteoarthritis; PBS = phosphate-buffered saline; RT-PCR = reverse transcriptase polymerase chain reaction; TUNEL = terminal dUTP nick-end labeling.

## Competing interests

The authors declare that they have no competing interests.

## Authors' contributions

LW carried out the study, analyzed the results, and drafted the manuscript. X-jS participated in the design and coordination of the study, and performed the characterization of cells. QC conceived the study, participated in its design, manuscript preparation, and coordination. All authors read and approved the final manuscript.

## Supplementary Material

Additional File 1 A figure showing anti-CD95 stimulated CD95L mRNA levels, as identified using the SYBR green real-time RT-PCR method. Briefly, 1 μg total RNA was reverse-transcribed into cDNA using iScripTM (Bio-Rad). Real-time quantitative PCR amplification was performed using QuantiTect SYBR Green PCR kit (Qiagen, Valencia, CA, USA) with DNA Engine Opticon 2 Continuous Fluorescence Detection System (MJ Research, Waltham, MA, USA). Click here for file

Additional File 2 A figure showing no significant difference in the phosphorylation of p38, ATF-2, c-Jun amino-terminal kinase (JNK), and extracellular signal-regulated kinase (ERK) between chondrocytes treated with or without an IgM isotype control antibody. Click here for file

Additional File 3 A figure showing that serum starvation will not effect cell death in an incubation period of 24 hours. Cell death was detected by trypan blue incorporation. Click here for file

## References

[B1] Bendele AM, Hulman JF (1988). Spontaneous cartilage degeneration in guinea pigs. Arthritis Rheum.

[B2] Vignon E, Arlot M, Patricot LM, Vignon G (1976). The cell density of human femoral head cartilage. Clin Orthop Relat Res.

[B3] Wei L, Brismar BH, Hultenby K, Hjerpe A, Svensson O (2003). Distribution of chondroitin 4-sulfate epitopes (2/B/6) in various zones and compartments of articular cartilage in guinea pig osteoarthrosis. Acta Orthop Scand.

[B4] Blanco FJ, Guitian R, Vazquez-Martul E, de Toro FJ, Galdo F (1998). Osteoarthritis chondrocytes die by apoptosis. A possible pathway for osteoarthritis pathology. Arthritis Rheum.

[B5] Hashimoto S, Ochs RL, Komiya S, Lotz M (1998). Linkage of chondrocyte apoptosis and cartilage degradation in human osteoarthritis. Arthritis Rheum.

[B6] Lotz M, Hashimoto S, Kuhn K (1999). Mechanisms of chondrocyte apoptosis. Osteoarthritis Cartilage.

[B7] Aigner T, Hemmel M, Neureiter D, Gebhard PM, Zeiler G, Kirchner T, McKenna L (2001). Apoptotic cell death is not a widespread phenomenon in normal aging and osteoarthritis human articular knee cartilage: a study of proliferation, programmed cell death (apoptosis), and viability of chondrocytes in normal and osteoarthritic human knee cartilage. Arthritis Rheum.

[B8] Hashimoto H, Tanaka M, Suda T, Tomita T, Hayashida K, Takeuchi E, Kaneko M, Takano H, Nagata S, Ochi T (1998). Soluble Fas ligand in the joints of patients with rheumatoid arthritis and osteoarthritis. Arthritis Rheum.

[B9] Kim HA, Lee YJ, Seong SC, Choe KW, Song YW (2000). Apoptotic chondrocyte death in human osteoarthritis. J Rheumatol.

[B10] Todd Allen R, Robertson CM, Harwood FL, Sasho T, Williams SK, Pomerleau AC, Amiel D (2004). Characterization of mature vs aged rabbit articular cartilage: analysis of cell density, apoptosis-related gene expression and mechanisms controlling chondrocyte apoptosis. Osteoarthritis Cartilage.

[B11] Chang L, Karin M (2001). Mammalian MAP kinase signalling cascades. Nature.

[B12] Paul A, Wilson S, Belham CM, Robinson CJ, Scott PH, Gould GW, Plevin R (1997). Stress-activated protein kinases: activation, regulation and function. Cell Signal.

[B13] Nebreda AR, Porras A (2000). p38 MAP kinases: beyond the stress response. Trends Biochem Sci.

[B14] Zhen X, Wei L, Wu Q, Zhang Y, Chen Q (2001). Mitogen-activated protein kinase p38 mediates regulation of chondrocyte differentiation by parathyroid hormone. J Biol Chem.

[B15] Pulsatelli L, Dolzani P, Piacentini A, Silvestri T, Ruggeri R, Gualtieri G, Meliconi R, Facchini A (1999). Chemokine production by human chondrocytes. J Rheumatol.

[B16] Chen Q, Johnson DM, Haudenschild DR, Goetinck PF (1995). Progression and recapitulation of the chondrocyte differentiation program: cartilage matrix protein is a marker for cartilage maturation. Dev Biol.

[B17] Mitrovic D, Quintero M, Stankovic A, Ryckewaert A (1983). Cell density of adult human femoral condylar articular cartilage. Joints with normal and fibrillated surfaces. Lab Invest.

[B18] Kuhn K, D'Lima DD, Hashimoto S, Lotz M (2004). Cell death in cartilage. Osteoarthritis Cartilage.

[B19] Kanbe K, Takagishi K, Chen Q (2002). Stimulation of matrix metalloprotease 3 release from human chondrocytes by the interaction of stromal cell-derived factor 1 and CXC chemokine receptor 4. Arthritis Rheum.

[B20] Kanbe K, Takemura T, Takeuchi K, Chen Q, Takagishi K, Inoue K (2004). Synovectomy reduces stromal-cell-derived factor-1 (SDF-1) which is involved in the destruction of cartilage in osteoarthritis and rheumatoid arthritis. J Bone Joint Surg Br.

[B21] Chen CT, Burton-Wurster N, Borden C, Hueffer K, Bloom SE, Lust G (2001). Chondrocyte necrosis and apoptosis in impact damaged articular cartilage. J Orthop Res.

[B22] Hashimoto S, Setareh M, Ochs RL, Lotz M (1997). Fas/Fas ligand expression and induction of apoptosis in chondrocytes. Arthritis Rheum.

[B23] Kaul M, Garden GA, Lipton SA (2001). Pathways to neuronal injury and apoptosis in HIV-associated dementia. Nature.

[B24] Schrantz N, Bourgeade MF, Mouhamad S, Leca G, Sharma S, Vazquez A (2001). p38-mediated regulation of an Fas-associated death domain protein-independent pathway leading to caspase-8 activation during TGFbeta-induced apoptosis in human Burkitt lymphoma B cells BL41. Mol Biol Cell.

[B25] Raingeaud J, Whitmarsh AJ, Barrett T, Derijard B, Davis RJ (1996). MKK3- and MKK6-regulated gene expression is mediated by the p38 mitogen-activated protein kinase signal transduction pathway. Mol Cell Biol.

[B26] Sano Y, Harada J, Tashiro S, Gotoh-Mandeville R, Maekawa T, Ishii S (1999). ATF-2 is a common nuclear target of Smad and TAK1 pathways in transforming growth factor-beta signaling. J Biol Chem.

[B27] Reimold AM, Grusby MJ, Kosaras B, Fries JW, Mori R, Maniwa S, Clauss IM, Collins T, Sidman RL, Glimcher MJ (1996). Chondrodysplasia and neurological abnormalities in ATF-2-deficient mice. Nature.

[B28] Faleiro L, Kobayashi R, Fearnhead H, Lazebnik Y (1997). Multiple species of CPP32 and Mch2 are the major active caspases present in apoptotic cells. EMBO J.

[B29] Huguier S, Baguet J, Perez S, van Dam H, Castellazzi M (1998). Transcription factor ATF2 cooperates with v-Jun to promote growth factor-independent proliferation *in vitro* and tumor formation in vivo. Mol Cell Biol.

[B30] Iannone F, De Bari C, Scioscia C, Patella V, Lapadula G (2005). Increased Bcl-2/p53 ratio in human osteoarthritic cartilage: a possible role in regulation of chondrocyte metabolism. Ann Rheum Dis.

[B31] Tetlow LC, Woolley DE (2003). Histamine stimulates the proliferation of human articular chondrocytes *in vitro* and is expressed by chondrocytes in osteoarthritic cartilage. Ann Rheum Dis.

