# Supplementary figures and images for: CD95-induced osteoarthritic chondrocyte apoptosis and necrosis: dependency on p38 mitogen-activated protein kinase
Source: Arthritis Res Ther. 2006 Jan 16;8(2):R37. doi: 10.1186/ar1891 (PMC1526592; doi:10.1186/ar1891)

**Relative transcript levels  
(arbitrary units, normalized to  
18S)**

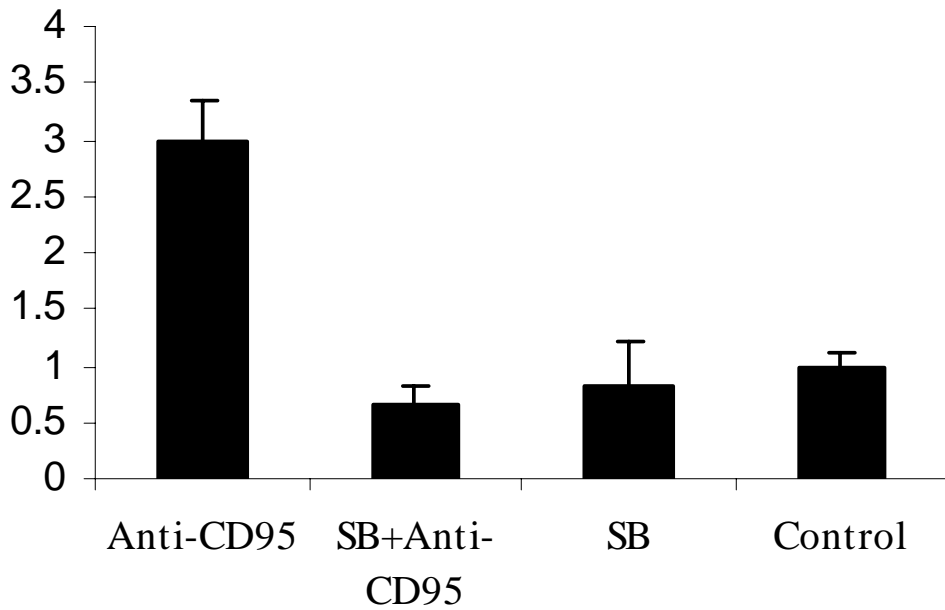

Supplement: Additional File 1 — A figure showing anti-CD95 stimulated CD95L mRNA levels, as identified using the SYBR green real-time RT-PCR method. Briefly, 1 μg total RNA was reverse-transcribed into cDNA using iScripTM (Bio-Rad). Real-time quantitative PCR amplification was performed using QuantiTect SYBR Green PCR kit (Qiagen, Valencia, CA, USA) with DNA Engine Opticon 2 Continuous Fluorescence Detection System (MJ Research, Waltham, MA, USA). [file ar1891-S1.pdf]

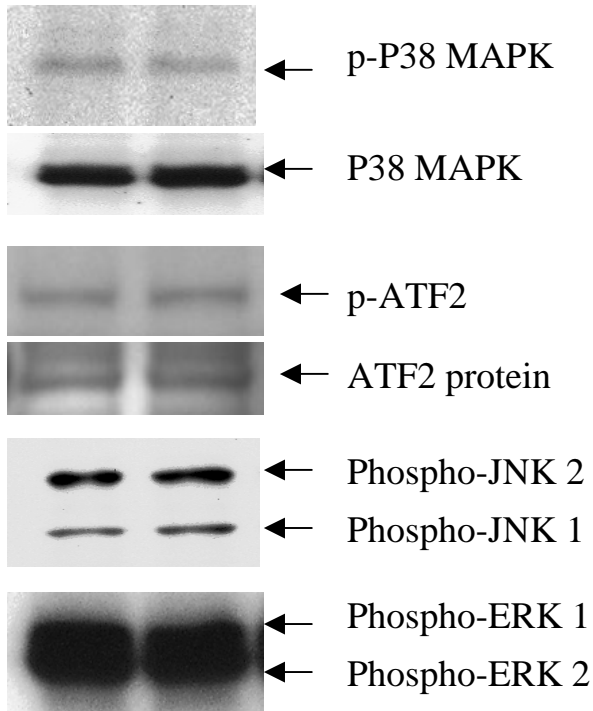

**Isotype control antibody**

-

+

Supplement: Additional File 2 — A figure showing no significant difference in the phosphorylation of p38, ATF-2, c-Jun amino-terminal kinase (JNK), and extracellular signal-regulated kinase (ERK) between chondrocytes treated with or without an IgM isotype control antibody. [file ar1891-S2.pdf]

Cell death detected by Trypan blue

5  
4  
3  
2  
1

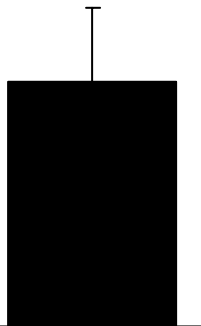

No Serum

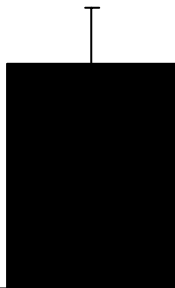

Serum

Supplement: Additional File 3 — A figure showing that serum starvation will not effect cell death in an incubation period of 24 hours. Cell death was detected by trypan blue incorporation. [file ar1891-S3.pdf]
